# Psychosocial Well-Being of Patients with Kidney Failure Receiving Haemodialysis during a Pandemic: A Survey

**DOI:** 10.3390/healthcare9081087

**Published:** 2021-08-23

**Authors:** Clare McKeaveney, Helen Noble, Claire Carswell, William Johnston, Joanne Reid

**Affiliations:** 1 School of Nursing and Midwifery, Queen’s University Belfast, 97 Lisburn Road, Belfast BT9 7BL, UK; C.McKeaveney@qub.ac.uk (C.M.); helen.noble@qub.ac.uk (H.N.); c.carswell@qub.ac.uk (C.C.); 2 Northern Ireland Kidney Patient Association, c/o Dialysis Unit, Belfast City Hospital, Lisburn Road, Belfast BT9 7AB, UK; willjohnston1@hotmail.co.uk; 3 Kidney Care, Alton GU34 1EF, UK

**Keywords:** kidney failure, haemodialysis, COVID-19, survey, well-being, mental health

## Abstract

Background: Living with kidney failure and undergoing hospital haemodialysis (HD) is associated with a high prevalence of mental health problems and poor quality of life. However, the COVID-19 pandemic has brought additional challenges for this patient population. Objectives: To understand the impact on mental health and well-being during the COVID-19 pandemic in people receiving HD. Methods: An online survey using a cross-sectional study design. Two validated assessment tools (General Health Questionnaire-12 (GHQ-12); Personal Wellbeing Index (PWI)), binary, Likert and free-text qualitative questions were included. Individuals with kidney failure receiving HD, over 18 years of age, were recruited online between July and August 2020. Results: Forty-four participants were recruited. Approximately, 54% of respondents were tested for COVID-19; however, no positive results were reported by patients or associated family members. Scores of GHQ-12 and PWI were compared with those from previous studies. Mental health distress was higher in prevalence (68.2%) and severity (M = 18.3) in this study when compared to that of the general population in Northern Ireland during COVID-19 (April 2020). In addition, well-being (M = 37.16, SD = 18.19) was poorer when compared to that of a pre-COVID-19 dialysis patient population. Conclusion: During the current pandemic, individuals receiving hospital HD have heightened mental health distress, and their well-being is impacted negatively. This study reinforces the need to provide appropriate psychosocial care as well as supportive interventions for mental distress to patients with kidney failure during and after the COVID-19 pandemic.

## 1. Introduction

Haemodialysis (HD) is the most frequently used renal replacement therapy for kidney failure (previously known as end-stage kidney disease; [1]) with approximately 37.4% of people in the United Kingdom receiving hospital HD treatment [2]. Patients receiving hospital HD experience a high burden of disease, arising from its chronic nature and protracted medical treatment [3]. Many patients receiving HD report poor quality of life scores in domains of lifestyle, social relationships and mental health [4,5,6,7]. Mental health conditions such as depression and anxiety are prevalent in renal disease patient populations [8]; higher in younger patients, in women and in black and minority ethnic groups [9]. Overall, living with kidney failure and undergoing hospital HD is extremely challenging [10].

The COVID-19 pandemic has brought additional challenges for this patient population, whereby patients receiving hospital HD are considered to be at higher risk of developing severe COVID-19 due to their immunocompromised status and frail condition [11]. Government guidance for the ‘clinically extremely vulnerable’ advises individuals such as those with kidney failure to take extra precautions during the peak of the pandemic, including shielding for a period of 12 weeks [12]. However, individuals attending hospital HD are unable to adhere to such strict guidelines due to twice or thrice weekly treatment. Inability to implement social distance during HD sessions places patients at further risk of viral transmission [13].

Previous pandemics have also shown individuals, including patients, are at an increased risk of developing post-traumatic stress symptoms. After the 2003 Severe Acute Respiratory Syndrome (SARS) epidemic, it was found that poor understanding of SARS and how it spread resulted in high rates of post-traumatic stress disorders [14]. This has additional implications for patients with kidney disease during the current pandemic, as mental health is an under-recognised and under-treated issue within this speciality. Poor mental health among people with kidney disease has wide-ranging implications, including increased risk of treatment non-compliance, hospitalisation and death [15]. Therefore, in light of the COVID-19 pandemic, patients with kidney failure are likely to be at an increased risk of such outcomes. Kidney Care UK administrated a COVID-19 survey exploring the impact of the pandemic on individuals with renal disease, showing significant disruption to people’s normal healthcare, negative consequences for mental health, distressing confusion over shielding and a lack of access to essential support [16]. However, no validated standardised questionnaires were used which can help to capture important mental health and quality of life dimensions for patients with kidney failure. 

There is an urgent need to understand the impact of the current pandemic on patients receiving hospital HD. This study aims to assess factors of mental health and well‐being using validated assessment tools, in addition to exploring patients’ experiences of shielding.

## 2. Materials and Methods

Design: This study was carried out using a cross-sectional study design. It was conducted over six weeks between July and August 2020. 

Sample: Individuals were recruited via an online survey link advertised within the Northern Ireland Kidney Patient Association (NIKPA) which has approximately 450 members. Those receiving hospital HD were included using the following inclusion criteria: patients with kidney failure receiving HD in Northern Ireland, over 18 years of age and ability to complete an online survey to capture qualitative and quantitative data. The study was approved by the Faculty of Medicine, Health and Life Sciences ethical committee (MHLS 20_77) within the host institution, and all methods were performed in accordance with the relevant guidelines and regulations. All participants completed an online informed consent form. 

Measures: This study combined validated questionnaires, demographic information and free-text qualitative questions via a web-based survey tool (Qualtrics^®^, Provo, Utah, USA). The study included author-designed binary, Likert and free-text qualitative questions to investigate experiences and personal concerns during the pandemic. In addition, two validated assessment tools were included. The General Health Questionnaire-12 (GHQ-12) was used to evaluate mental health status in the present study. It is a highly reliable and validated screening instrument for mental health [17]. The GHQ-12 measures 12 psychological symptoms of mental distress over the past few weeks. The GHQ-12 score includes factors associated with social dysfunction (items 1, 3, 4, 7, 8 and 12), anxiety and depression (items 2, 5, 6 and 9) and loss of confidence (items 10 and 11). Each item of the GHQ-12 is rated on a 4-point Likert scale (0-1-2-3) with higher scores indicating greater mental distress (range 0–36). A bi-modal score (using 0-0-1-1) can also be used to provide the percentage proportion of the population experiencing mental health distress [18,19]. 

The Personal Wellbeing Index (PWI) investigated patient well-being and contains seven items of satisfaction, each one corresponding to a quality-of-life domain, i.e., standard of living, health, achieving in life, relationships, safety, community connectedness and future security [20]. PWI is a highly validated and reliable tool [21]. Respondents were asked how satisfied they were with their life, reporting a score from zero to 10. Zero means no satisfaction at all, and 10 means completely satisfied, whereby higher scores indicate greater life satisfaction. All scores were transformed from a 0–10 scale to a 0–100 scale.

Analysis: Descriptive statistics were provided for demographic information including a narrative description of binary and Likert responses. Mean and standard deviations (+SD scores) were calculated for standardised measures of mental health (GHQ-12) and well-being (PWI). A t-test was conducted using GraphPad Prism 9^®^ (GraphPad software, San Diego, CA, USA) for GHQ-12 mean scores (*p* < 0.05). Qualitative open-text responses were analysed using thematic analysis [22]. 

## 3. Results

Data from both qualitative and quantitative analysis are presented together in a mixed-method approach. This includes the use of frequencies, standardised assessment scores and open-ended questions to describe and understand the impact of COVID-19 in people receiving hospital HD [23]. Forty-four participants who were receiving HD in Northern Ireland completed this online study (see Table 1). The sample included 26 males (59.1%) and 17 females (38.6%; *n* = 1 not identified). The majority of respondents were ≥45 years old (*n* = 32), with the largest cohort ≥ 65 years old (*n* = 16), which is representative of UK patients (median age of HD start is 67.4; [2]). The majority of respondents were white (93.2%), married (52.3%), attending HD three times a week (81.8%) and not employed (68.2%).

Approximately 54% of respondents had been tested for coronavirus since the beginning of the pandemic (Table 2); however, no positive tests were reported. No family members or loved ones had been tested for coronavirus. Fifteen (34%) respondents reported they had attended a facility that had cared for patients with known or suspected COVID-19, yet 31% were unsure of any positive diagnoses within their respective clinic. 

### 3.1. Open-Text Responses 

Further difficulties were highlighted within free-text responses. Shielding led to significant feelings of loneliness; “[shielding causes] sorrow”, “feeling forgotten about” and “I am finding the loneliness difficult” as normal life for patients ceased, “unable to work or go out with my girlfriend” and “isolated from family.” Experiences of shielding also led to significant feelings of anxiety, whereby respondents felt “forgotten about” and “very vulnerable.” Respondents also expressed growing symptoms of depression, “…it is making me feel more depressed” and feelings of being “…fed up.” Patients reported not feeling confident when in the general public “not many people sticking to the guidelines in public…”, and this led to additional burdens on the patient, “…decided to drive myself [to dialysis].” Conversely, shielding was associated with feelings of “safety”; however, it also had consequences, “I feel much safer shielding, but it is making me feel more depressed” as well as anxieties about “…travelling to hospital.” 

Respondents highlighted worries over disruption to their healthcare, “hospital appointments being stopped and holding things back.” Many respondents expressed struggling with worries about the future, “I am worried it will have to be like this forever” and the uncertainty about their future, “not being able to go out is difficult but I despair about the future.” 

The majority of respondents had experienced changes to their dialysis treatment since the pandemic commenced, whereby being at the hospital “…made [patients] feel even more isolated” and “…uneasy around other people.” This included patients reporting “less interaction at first with staff” and staff were “[stricter]” within the HD units. The majority of respondents reported they “confidently understand” their healthcare guidelines on COVID-19; however, patients expressed specific gaps in knowledge and needed “more information.”

### 3.2. Mental Health

Table 3 shows the total average score of the GHQ-12 was 18.3. Using the bi-modal assessment and cut-off point (≥4; [19]), the majority of those attending hospital for HD treatment demonstrated mental distress (68.2%). Participants tended to have poorer responses on positive items (see Supplementary Table S1 for breakdown). Respondents indicated they did not feel they were ‘playing a useful part’ in life, ‘capable of making decisions about things’ or ‘able to enjoy…normal day-to-day activities’, “less than usual” or “much less than usual”. 

### 3.3. Well-Being

The total average score of the PWI was 37.16 (SD = 18.19; Table 4), which was significantly lower than that of a comparative pre-COVID-19 dialysis sample [24] (M = 64.72, SD = 19.17; t = 8.587, df = 212, *p* < 0.01), indicating poorer well-being. Scores for all items were lower than those of the comparative group, in particular, statistically significant differences were reported for personal relationships (M = 65.45, SD = 34.27; t = 2.052, df = 212, *p* = 0.04), personal safety (M = 57.95, SD = 32.82; t = 3.659, df = 212, *p* < 0.01), community connectedness (M = 50.00, SD = 33.06; t = 3.104, df = 212, *p* < 0.01)) and future security (M = 45.91, SD = 28.31; t = 3.864, df = 212, *p* < 0.01). 

## 4. Discussion

The overarching aim of this study was to understand the impact of COVID-19 in patients with kidney failure receiving HD, by assessing factors of mental health, well-being and reporting on experiences of shielding. We found heightened mental health distress and reduced well-being. Qualitative findings help to emphasise the psychological, social and emotional distress experienced during the pandemic. These outcomes provide an early understanding of the unique experiences of this patient population within the United Kingdom. Similar findings were also reported by Lee and colleagues [25] who found an adverse psychosocial impact of COVID-19 on patients with kidney failure receiving haemodialysis within the United States.

Compared to a general population sample in April 2020 from Northern Ireland, the GHQ-12 scores were substantially worse for the patients receiving HD in this study [19]. The overall mean scores of mental distress were substantially lower when compared to those of the current sample of patients receiving hospital HD. In addition, factors associated with ‘social dysfunction’ (e.g., able to concentrate’, ‘playing a useful part’, ‘capable of making decisions about things’ and ‘able to enjoy your normal day-to-day activities’) tended to be poorer. This is not surprising, as social isolation is a common difficulty in patients with kidney disease [26]. In particular, patients with kidney failure receiving HD, face increased social isolation and support difficulties due to the intensive and protracted nature of dialysis treatment [8], having less time for employment, hobbies and other social activities [7]. Conversely, the COVID-19 pandemic may contribute to far greater levels of depression and anxiety [27] and progressive social withdrawal [28] in this patient population. 

PWI scores also highlight reduced well-being in this patient sample. Patients receiving HD tend to have significantly impacted quality of life compared to those receiving other renal replacement therapies; however, when compared to a pre-COVID-19 sample [24], the overall score as well as individual items on the PWI, suggest the current pandemic may be further impacting on quality of life in patients with kidney failure receiving HD. Caution should be applied, as comparisons include a similar but Australian sample; however, qualitative data further demonstrates how shielding and self-isolation during the pandemic have contributed to reduced well-being including experiences of social anxiety, uncertainty about the future and feelings of loneliness. 

It has been long recognised that patients receiving HD experience reduced mental health and quality of life outcomes [29,30,31], yet little evidence currently exists relating to effective interventions [32]. Now more than ever, enhancing opportunities for psychosocial support is necessary to improve the mental health and well-being of those attending hospital HD. The role of non-pharmacological management of mental health conditions is gaining support within renal medicine [33], but this is slow to fruition within practice. Recent evidence shows perceived social support is also an important factor in patients’ ability to cope with their illness and can improve well-being [34]. Therefore, novel interventions that maintain or improve social well-being, including social support, social participation and relationships, may help improve quality of life for this patient population. 

Research on peer mentor programs is growing with positive outcomes in patients with kidney failure [35,36]. Patient-to-patient peer mentor support aims to provide a platform for shared experiences, emotional and educational support as well as a foundation for social activities [37]. Such interventions could help to address the psychological, social and emotional impact of the COVID-19 pandemic identified in this study. Formally embedding such an approach into clinical practice would also help to ensure maximum optimisation and prioritisation for patients with kidney failure receiving hospital HD during the current pandemic and beyond [38].

Future research should include an ethnically representative sample of the wider UK renal patient population (approximately 30% are minority groups; [2]). The modest sample size and sampling framework limit the generalisability of the results, particularly for those without access or the ability to complete the online survey. However, during lockdown, this patient perspective was at risk of being overlooked. 

## 5. Implications for Clinical Practice

Undoubtedly, the current pandemic has fast-tracked the urgency for future research to understand psychological distress and social dysfunction in kidney failure. Patients with kidney failure undergoing hospital HD should be screened regularly to assess for mental health symptoms, and prompt referral and treatment must be initiated where required. By addressing needs, appropriate policies and supportive interventions that seek to prevent and reduce perceived isolation and psychological distress can be developed to monitor and manage patients at significant risk of reduced mental health and well-being. 

## 6. Conclusions

This study provides a unique snapshot of individuals attending hospital HD during the current COVID-19 pandemic, using validated assessments measures. There is an urgent need to support mental health and the development of supportive interventions that may help improve psychosocial care in patients with kidney failure, particularly during and after the COVID-19 pandemic. 

## Figures and Tables

**Table 1:** Sociodemographic variables.

Demographic Item	N	%
Gender		
Male	26	59.1%
Female	17	38.6%
Prefer not to say	1	2.3%
Marital status		
Single	12	27.3%
Married/co-habiting	23	52.3%
Widowed	9	20.4%
Employed		
Yes	14	31.8%
No	30	68.2%
Ethnicity		
White	41	93.2%
Other	3	6.8%
Age		
25–34	8	18.2%
35–44	4	9.0%
45–54	8	18.2%
55–64	8	18.2%
65+	16	36.4%
Education		
Further/university level	8	18.2%
Secondary level	28	63.6%
Primary level	8	18.2%
Caring responsibilities		
Yes	2	4.6%
No	42	95.4%
Transplant before		
Yes	10	22.7%
No	34	77.3%
Haemodialysis sessions		
4 × week	6	13.6%
3 × week	36	81.8%
≤ 2 × week	2	4.6%

**Table 2:** COVID-19 testing experiences.

	Yes	No	Unsure
Tested for COVID-19	54%	46%	-
Loved ones tested for COVID-19	0%	100%	-
Attended a facility that cared for patients with known or suspected COVID-19	34%	35%	31%

**Table 3:** Standardised measure of General Health Questionnaire-12 (GHQ-12).

	Kidney Failure GHQ-12 Total Score (95% CI)	General Population GHQ-12 Total Score during COVID-19 * (95% CI)
GHQ-12 total score	18.3 (15.8–20.9)	12.5 (11.5–13.5)
Proportion with significant level of mental distress	68.2%	28.5%

* Northern Ireland general population sample GHQ-12 during the COVID-19 pandemic recorded in April 2020 [19]; CI = Confident Interval.

**Table 4:** Standardised measure of Perceived Wellbeing Index (PWI).

Item	Kidney Failure during COVID-19 Mean (SD)	Kidney Failure Pre-COVID-19 * Mean (SD)
Standard of Living	62.73 (28.15)	68.72 (22.42)
Personal Health	43.18 (24.38)	47.67 (24.36)
Achieving in Life	46.36 (26.68)	54.34 (26.43)
Personal Relationships	65.45 (34.27) **	74.97 (25.39)
Personal Safety	57.95 (32.82) **	73.55 (22.87)
Community-Connectedness	50.00 (33.06) **	64.85 (26.93)
Future Security	45.91 (28.31) **	63.78 (27.09)
Total score	37.16 (18.19) **	64.72 (19.17)

All scores were transformed from a 0–10 scale to a 0–100 scale. * Kidney failure sample taken before COVID-19 [24]; ** Significance = *p* < 0.05.

## Data Availability

The data presented in this study are available on request from the corresponding author.

## Supplementary Materials

The following is available online at https://www.mdpi.com/article/10.3390/healthcare9081087/s1, Table S1: Standardised measure of GHQ-12 with item and frequency breakdown.

## References

[1] Levey A.S., Eckardt K.U., Dorman N.M., Christiansen S.L., Cheung M., Jadoul M., Winkelmayer W.C. (2020). Nomenclature for kidney function and disease-executive summary and glossary from a Kidney Disease: Improving Global Outcomes (KDIGO) consensus conference. Eur. Heart J..

[2] UK Renal Registry (2020). UK Renal Registry Summary of Annual Report—Analyses of Adult Data to the End of 2018.

[3] Kalfoss M., Schick-Makaroff K., Molzahn A.E. (2019). Living with Chronic Kidney Disease: Illness Perceptions, Symptoms, Coping, and Quality of Life. Nephrol. Nurs. J..

[4] Brown E.A., Zhao J., McCullough K., Fuller D.S., Figueiredo A.E., Bieber B., Finkelstein F.O., Shen J., Kanjanabuch T., Kawanishi H. (2021). Burden of Kidney Disease, Health-Related Quality of Life, and Employment Among Patients Receiving Peritoneal Dialysis and In-Center Hemodialysis: Findings From the DOPPS Program. Am. J. Kidney Dis..

[5] Nataatmadja M., Evangelidis N., Manera K.E., Cho Y., Johnson D.W., Craig J.C., Baumgart A., Hanson C.S., Shen J., Guha C. (2021). Perspectives on mental health among patients receiving dialysis. Nephrol. Dial. Transplant..

[6] Neumann D., Lamprecht J., Robinski M., Mau W., Girndt M. (2018). Social relationships and their impact on health-related outcomes in peritoneal versus haemodialysis patients: A prospective cohort study. Nephrol. Dial. Transplant..

[7] Dąbrowska-Bender M., Dykowska G., Żuk W., Milewska M., Staniszewska A. (2018). The impact on quality of life of dialysis patients with renal insufficiency. Patient Prefer. Adherence.

[8] Goh Z., Griva K. (2018). Anxiety and depression in patients with end-stage renal disease: Impact and management challenges—A narrative review. Int. J. Nephrol. Renov. Dis..

[9] Damery S., Sein K., Nicholas J., Baharani J., Combes G. (2019). The challenge of managing mild to moderate distress in patients with end stage renal disease: Results from a multi-centre, mixed methods research study and the implications for renal service organisation. BMC Health Serv. Res..

[10] Weinberg M.K., Bennett P., Cummins R. (2015). Validation of the Personal Wellbeing Index for People with End Stage Kidney Disease. Appl. Res. Qual. Life.

[11] Gansevoort R.T., Hilbrands L.B. (2020). CKD is a key risk factor for COVID-19 mortality. Nat. Rev. Nephrol..

[12] Department of Health and Social Care (2020). Guidance on Shielding and Protecting People Who Are Clinically Extremely Vulnerable from COVID-19. https://www.gov.uk/government/publications/guidance-on-shielding-and-protecting-extremely-vulnerable-persons-from-covid-19/guidance-on-shielding-and-protecting-extremely-vulnerable-persons-from-covid-19.

[13] Ikizler T.A., Kliger A.S. (2020). Minimizing the risk of COVID-19 among patients on dialysis. Nat. Rev. Nephrol..

[14] Dutheil F., Mondillon L., Navel V. (2020). PTSD as the second tsunami of the SARS-Cov-2 pandemic. Psychol. Med..

[15] Fischer M.J., Porter A.C., Lash J.P. (2013). Treatment of Depression and Poor Mental Health among Patients Receiving Maintenance Dialysis: Are There Options Other Than a Pill or a Couch?. Am. J. Kidney Dis..

[16] Sharp S., Loud F. (2020). ‘Worried sick’—The impact of COVID-19 on people living with kidney disease: Findings from a patient survey. J. Kidney Care.

[17] Goldberg D.P. (1988). User’s Guide to the General Health Questionnaire.

[18] Goldberg D.P., Gater R., Sartorius N., Ustun T.B., Piccinelli M., Gureje O., Rutter C. (1997). The validity of two versions of the GHQ in the WHO study of mental illness in general health care. Psychol. Med..

[19] Pierce M., Hope H., Ford T., Hatch S., Hotopf M., John A., Kontopantelis E., Webb R., Wessely S., McManus S. (2020). Mental health before and during the COVID-19 pandemic: A longitudinal probability sample survey of the UK population. Lancet Psychiatry.

[20] Cummins R.A., Eckersley R., Pallant J., Van Vugt J., Misajon R. (2003). Developing a National Index of Subjective Wellbeing: The Australian Unity Wellbeing Index. Soc. Indic. Res..

[21] Lau A.L.D., Cummins R., McPherson W. (2005). An Investigation into the Cross-Cultural Equivalence of the Personal Wellbeing Index. Soc. Indic. Res..

[22] Braun V., Clarke V. (2006). Using thematic analysis in psychology. Qual. Res. Psychol..

[23] Brewer J., Hunter A. (2006). Foundations of Multimethod Research: Synthesizing Styles.

[24] Bennett P.N., Weinberg M.K., Bridgman T., Cummins R. (2015). The happiness and subjective well-being of people on haemodialysis. J. Ren. Care.

[25] Lee J., Steel J., Roumelioti M.-E., Erickson S., Myaskovsky L., Yabes J.G., Rollman B.L., Weisbord S.D., Unruh M., Jhamb M. (2020). Psychosocial Impact of COVID-19 Pandemic on Patients with End-Stage Kidney Disease on Hemodialysis. Kidney360.

[26] Moorthi R.N., Latham-Mintus K. (2019). Social isolation in chronic kidney disease and the role of mobility limitation. Clin. Kidney J..

[27] Palmer S., Vecchio M., Craig J., Tonelli M., Johnson D.W., Nicolucci A., Pellegrini F., Saglimbene V., Logroscino G., Fishbane S. (2013). Prevalence of depression in chronic kidney disease: Systematic review and meta-analysis of observational studies. Kidney Int..

[28] Porcelli S., Van Der Wee N., van der Werff S., Aghajani M., Glennon J.C., van Heukelum S., Mogavero F., Lobo A., Olivera F.J., Lobo E. (2018). Social brain, social dysfunction and social withdrawal. Neurosci. Biobehav. Rev..

[29] Sapilak B.J., Kurpas D., Steciwko A.F., Melon M. (2006). Importance of hemodialysed patients’ quality of life? Based on 3-years observation. Probl. Lek. Med. Probl..

[30] Davison S.N., Jhangri G.S. (2010). Impact of Pain and Symptom Burden on the Health-Related Quality of Life of Hemodialysis Patients. J. Pain Symptom Manag..

[31] Jankowska-Polańska B., Uchmanowicz I., Wysocka A., Lomper K., Fal A.M., Uchmanowicz B. (2016). Factors affecting the quality of life of chronic dialysis patients. Eur. J. Public Health.

[32] Sharma S., Green T., Alexander K.E., Bonner A. (2020). Educational or behavioural interventions for symptoms and health--related quality of life in adults receiving haemodialysis: A systematic review. J. Ren. Care.

[33] Ng C.Z., Tang S.C.W., Chan M., Tran B.X., Ho C.S., Tam W.W., Ho R.C. (2019). A systematic review and meta-analysis of randomized controlled trials of cognitive behavioral therapy for hemodialysis patients with depression. J. Psychosom. Res..

[34] Alshraifeen A., Al Rawashdeh S., Alnuaimi K., Alzoubi F., Tanash M., Ashour A., Al Hawamdih S., Al Ghabeesh S. (2020). Social support predicted quality of life in people receiving haemodialysis treatment: A cross-sectional survey. Nurs. Open.

[35] Bennett P.N., Russell J.S.C., Atwal J., Brown L., Schiller B. (2018). Patient-to-patient peer mentor support in dialysis: Improving the patient experience. Semin. Dial..

[36] Willis M.A., Hein L.B., Hu Z., Saran R., Argentina M., Bragg-Gresham J., Krein S.L., Gillespie B., Zheng K., Veinot T.C. (2021). Feeling better on hemodialysis: User-centered design requirements for promoting patient involvement in the prevention of treatment complications. J. Am. Med. Inform. Assoc..

[37] Think Kidneys (2016). Transforming Participation in Chronic Kidney Disease (CKD) Interventions Toolkit. https://www.thinkkidneys.nhs.uk/ckd/wp-content/uploads/sites/4/2017/05/Interventions-Toolkit-FINAL-2.pdf.

[38] Wood E., Trasolini A., Thomas N. (2021). Barriers and facilitators to implementing and sustaining peer support in kidney. J. Ren. Care.

