# Supplementary material for: Psychosocial Well-Being of Patients with Kidney Failure Receiving Haemodialysis during a Pandemic: A Survey
Source: Healthcare (Basel). 2021 Aug 23;9(8):1087. doi: 10.3390/healthcare9081087 (PMC8392847; doi:10.3390/healthcare9081087)
Supplement: Supplementary file 1 [file healthcare-09-01087-s001.zip › healthcare-1284553-supplementary.pdf]

healthcare

MDPI

Supplementary file

Psychosocial Wellbeing of Patients Receiving Haemodialysis during a Pandemic: A Multi-Method Approach

Table S1. Standardised measure of GHQ-12 with item and frequency breakdown.

General Health Questionaire-12		Response frequencies			
		0 ☺	1	2	3 ☹
1	Able to concentrate	11.4%	31.8%	34.1%	22.7%
2	Lost much sleep	18.2%	40.9%	34.1%	6.8%
3	Playing a useful part	4.5%	0.0%	72.8%	22.7%
4	Capable of making decisions	6.8%	0.0%	75.0%	18.2%
5	Under stress	4.5%	54.6%	31.8%	9.1%
6	Could not overcome difficulties	25.0%	27.3%	34.1%	13.6%
7	Enjoy your day-to-day activities	4.5%	29.5%	31.9%	34.1%
8	Face up to problems	0.0%	61.4%	25.0%	13.6%
9	Feeling unhappy and depressed	22.7%	20.5%	47.7%	9.1%
10	Losing confidence	29.5%	31.8%	20.5%	18.2%
11	Thinking of self as worthless	56.8%	20.5%	9.1%	13.6%
12	Feeling reasonably happy	4.5%	59.2%	22.7%	13.6%
